# Cascading After Peridiagnostic Cancer Genetic Testing: An Alternative to Population-Based Screening

**DOI:** 10.1200/JCO.19.02010

**Published:** 2020-01-10

**Authors:** Kenneth Offit, Kaitlyn A. Tkachuk, Zsofia K. Stadler, Michael F. Walsh, Hector Diaz-Zabala, Jeffrey D. Levin, Zoe Steinsnyder, Vignesh Ravichandran, Ravi N. Sharaf, Melissa K. Frey, Steven M. Lipkin, Mark E. Robson, Jada G. Hamilton, Joseph Vijai, Semanti Mukherjee

**Affiliations:** ^1^ Clinical Genetics Service, Department of Medicine, Memorial Sloan Kettering Cancer Center; and Program in Cancer Biology and Genetics, Sloan Kettering Institute, New York, NY; ^2^ Department of Psychiatry and Behavioral Sciences, Memorial Sloan Kettering Cancer Center, New York, NY; ^3^ Breast Medicine Service, Department of Medicine, Memorial Sloan Kettering Cancer Center, New York, NY; ^4^ Weill Cornell College of Medicine, Cornell University, New York, NY

## Abstract

**PURPOSE:**

Despite advances in DNA sequencing technology and expanded medical guidelines, the vast majority of individuals carrying pathogenic variants of common cancer susceptibility genes have yet to be identified. An alternative to population-wide genetic screening of healthy individuals would exploit the trend for genetic testing at the time of cancer diagnosis to guide therapy and prevention, combined with augmented familial diffusion or “cascade” of genomic risk information.

**METHODS:**

Using a multiple linear regression model, we derived the time interval to detect an estimated 3.9 million individuals in the United States with a pathogenic variant in 1 of 18 cancer susceptibility genes. We analyzed the impact of the proportion of incident patients sequenced, varying observed frequencies of pathogenic germline variants in patients with cancer, differential rates of diffusion of genetic information in families, and family size.

**RESULTS:**

The time to detect inherited cancer predisposing variants in the population is affected by the extent of cascade to first-, second-, and third-degree relatives (FDR, SDR, TDR, respectively), family size, prevalence of mutations in patients with cancer, and the proportion of patients with cancer sequenced. In a representative scenario, assuming a 7% prevalence of pathogenic variants across cancer types, an average family size of 3 per generation, and 15% of incident patients with cancer in the United States undergoing germline testing, the time to detect all 3.9 million individuals with pathogenic variants in 18 cancer susceptibility genes would be 46.2, 22.3, 13.6, and 9.9 years if 10%, 25%, 50%, and 70%, respectively, of all FDR, SDR, and TDR were tested for familial mutations.

**CONCLUSION:**

Peridiagnostic and cascade cancer genetic testing offers an alternative strategy to achieve population-wide identification of cancer susceptibility mutations.

## INTRODUCTION

More than 2 decades after the discovery of genetic variants conferring substantial risks for breast, ovarian, prostate, colon, and other malignancies, and despite advances in DNA sequencing technologies, it has become increasingly clear that the promise of genomics as a tool for cancer prevention has yet to be realized.^1^ Fewer than 1 in 5 individuals with a family history of breast or ovarian cancer who meet established criteria for genetic testing have received it, and most have never discussed their hereditary cancer risk with a health care provider.^2^ For Lynch syndrome, the most common colon cancer predisposition syndrome, only 1 in 4 individuals who met criteria were screened in the Cancer Research Network,^3^ and 64%-85% of Community Hospital Cancer Programs do not screen for this syndrome.^4^

The scale of these shortcomings is measured in lives lost because for Lynch syndrome, as for hereditary breast and ovarian cancer, medical or surgical interventions have been shown to decrease mortality.^1,5^ To address these challenges more broadly, there have been renewed calls for population-wide testing for *BRCA* and other genes,^6,7^ and in the United States and abroad, efforts have begun to sequence the DNA of large cohorts to screen for a broad spectrum of inherited risks.^8,9^ At the same time, for-profit companies have sought to market genomic testing outside of the traditional medical context, despite public and professional diminished trust in the accuracy and privacy of direct-to-consumer genetic testing.^10-14^

Because population-based approaches to genomic screening remain costly and involve challenges in high through-put sequencing, obtaining informed consent, and interpretation of genomic variants,^15,16^ alternative strategies have been proposed, including testing isolated populations at higher risk for cancer,^17,18^ as well as testing family members of those found to harbor pathogenic variants (mutations) in disease predisposition genes (Data Supplement). An emerging strategy, emphasized in this article, takes its origin from the increasing use of genetic testing at the time of a diagnosis of cancer, affecting both preventive and therapeutic management.^19,20^ Rapidly expanding peridiagnostic analysis of patients with cancer has the potential to allow physician-facilitated family outreach to disseminate risk information and promote cancer prevention. We seek to model various parameters that would affect the time needed to identify *all* individuals heterozygous for pathogenic variants of clinically actionable cancer susceptibility genes in the United States through a strategy of peridiagnostic cancer genetic testing and family diffusion.

## METHODS

To model the time course of familial transmission of testing for a pathogenic variant after peridiagnostic tumor-normal or germline panel testing, we assumed various rates of detection of pathogenic variants in probands and diffusion of this information in families, starting with 1.7 million patients with cancer diagnosed per year in the United States,^21^ assuming these to be among unrelated individuals, with a fraction of these patients offered tumor-normal or germline sequencing for a panel of cancer predisposition genes. For this analysis, a subset of 18 “clinically actionable” genes (Table 1) was chosen from a larger group of genes used in a prior analysis.^20^ Clinical actionability of pathogenic variants was defined by evidence for their utility in cancer prevention and/or potential utility as therapeutic targets.^20^ For this analysis, *CHEK2, MUTYH, CDKN2A*, and *NBN* were not included because of low penetrance of some common variants (*CHEK2*) or uncertain clinical actionability. However, all “high penetrance” genes proposed in a recent population-screening proposal^7^ were included.

**TABLE 1.**
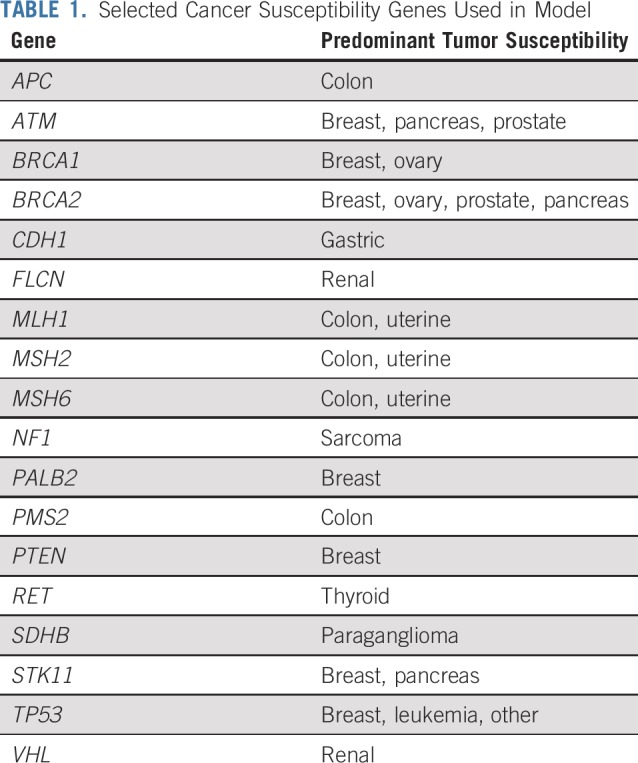
Selected Cancer Susceptibility Genes Used in Model

We assumed the prevalence of germline mutations in patients sequenced ranged from 5% to 15%.^20-22^ We next considered extended families across 3 generations with 2 to 4 offspring per generation^23^ (Data Supplement), with communication progressing from proband to first-, second-, and third-degree relatives (FDR, SDR, TDR, respectively) and then in successive years to FDR, SDR, and TDR of those contacted the prior year (Data Supplement). We projected that from 0% to 75% of relatives potentially heterozygous for an inherited mutation would be tested. We estimated the number of individuals carrying pathogenic variants already identified (Data Supplement). Driven by “cascade” testing of incident patients with cancer, we measured the time to pass a calculated threshold of 3.9 million individuals older than 25 years of age in the United States, representing the number heterozygous for pathogenic variants in this panel of 18 genes, as directly estimated by frequencies in the ExAC resource (Data Supplement). We assumed a steady state of mutation carriers with countervailing influences of compromised fitness, death due to hereditary cancer, and preimplantation genetic diagnosis, balanced by the birth and immigration of new carriers of pathogenic variants.

**FIG 1.**
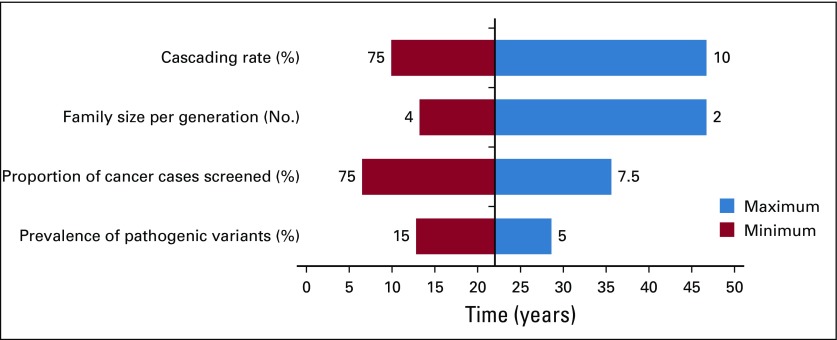
Tornado plot depicting univariate sensitivity analysis performed using baseline model: 7% prevalence of pathogenic variants across cancer types, an average family size of 3 per generation, 15% of incident patients with cancer undergoing germline testing, and 25% first-degree relatives, 25% second-degree relatives, and 25% third-degree relatives cascading, the time to detect 3.9 million individuals with a germline cancer susceptibility mutation was 22.2 years. The plot shows the effect on the output (number of years) of varying each input variable at a time, keeping all the other input variables at their baseline value. The cascading rate here is defined by the proportion of first-degree, second-degree, and third-degree relatives transmitting information, ranging from 10% to 75%; family size per generation refers to the number of siblings per generation, ranging from 2 to 4; proportion of patients with cancer screened refers to the proportion of incident patients with cancer subjected to germline sequencing of 18 selected genes, ranging from 7.5% to 75%; prevalence of pathogenic mutations refers to the prevalence of pathogenic variants of 18 cancer susceptibility genes in patients with cancer sequenced, ranging from 5% to 15%. The upper and lower bounds for each variable are labeled in the tornado plot.

We performed a sensitivity analysis on time (in years) to reach a threshold of detection of 3.9 million heterozygotes for these pathogenic variants by varying parameters, including the proportion of patients with cancer in the United States tested (range, 7.5%-75%), the proportion of patients with cancer with germline mutations (5%-15%), and the proportion of FDR, SDR, and TDR tested for familial pathogenic variants (0% to 75%). Multiple linear regression was performed in RStudio version 1.0.143^24^ on log-transformed response variables to define significant determinants/parameters and the magnitude of their effects (Data Supplement). A tornado plot was generated for the graphic representation of the degree to which the number of years (the result) was sensitive to the specific parameters, thus depicting the univariate sensitivity analysis (Fig 1).

## RESULTS

**FIG 2.**
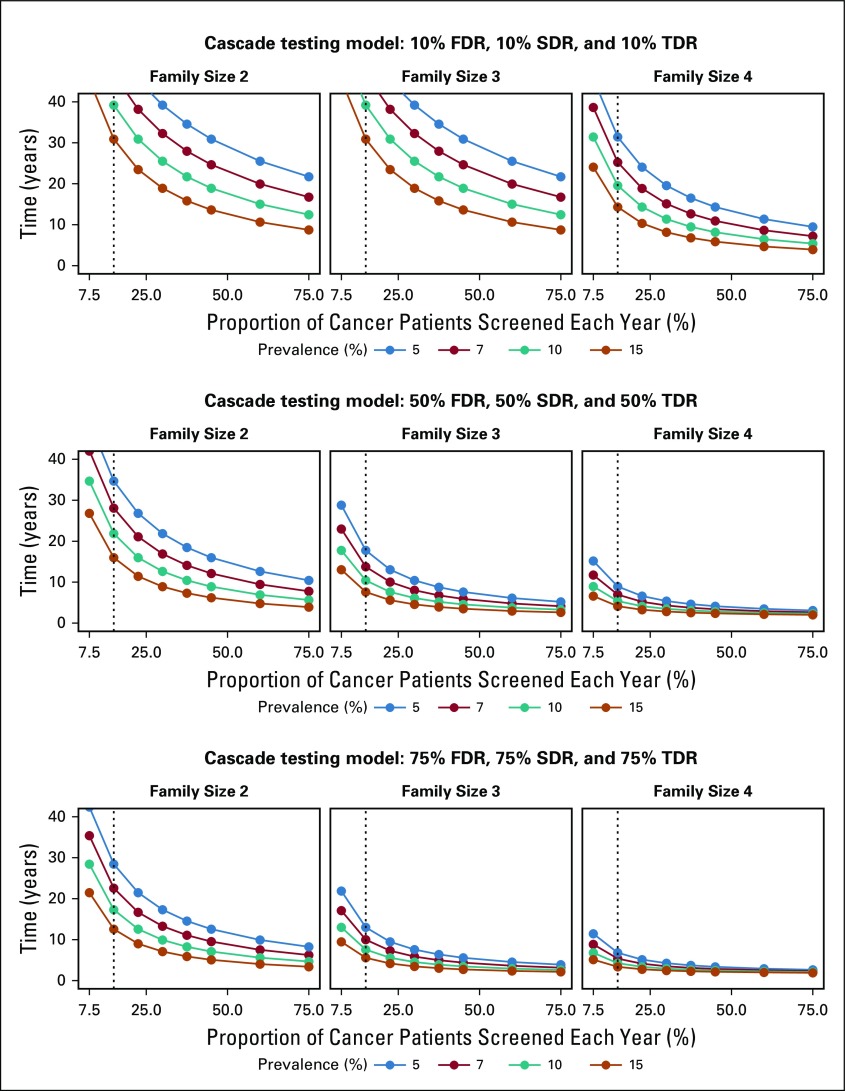
Sensitivity analysis; Prevalence (%), patients with mutations of 18 cancer susceptibility genes; Proportion, of 1.7 million incident cancers sequenced; Years to detect 3.9 million mutation carriers. FDR, first-degree relatives; SDR, second-degree relatives; TDR, third-degree relatives.

**FIG 3.**
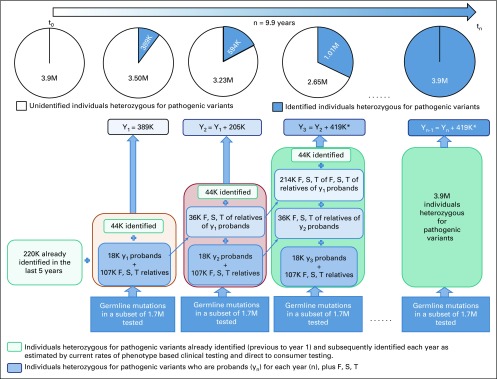
Diagram of peridiagnostic genetic testing and familial diffusion of information under the assumptions of a family size of 3 siblings per generation, 15% of patients with cancer receiving germline genetic testing at the time of diagnosis, a 7% frequency of pathogenic mutations found at the time of testing, and 70% diffusion of familial genetic information to first-degree relatives (F), second-degree relatives (S), and third-degree relatives (T). In this example, it is assumed that 220,000 carriers of pathogenic variants have been identified in the United States, with 44,000 additional individuals identified each year by virtue of expanded commercial clinical and direct-to-consumer testing. Y_n_ represents the cumulative number of individuals with pathogenic variants identified each year. Under these assumptions, the time to detect all 3.9 million individuals with pathogenic variants in the United States is 9.9 years. (*) 419K heterozygous for pathogenic variants identified each year after Y_3_ (Data Supplement).

**FIG 4.**
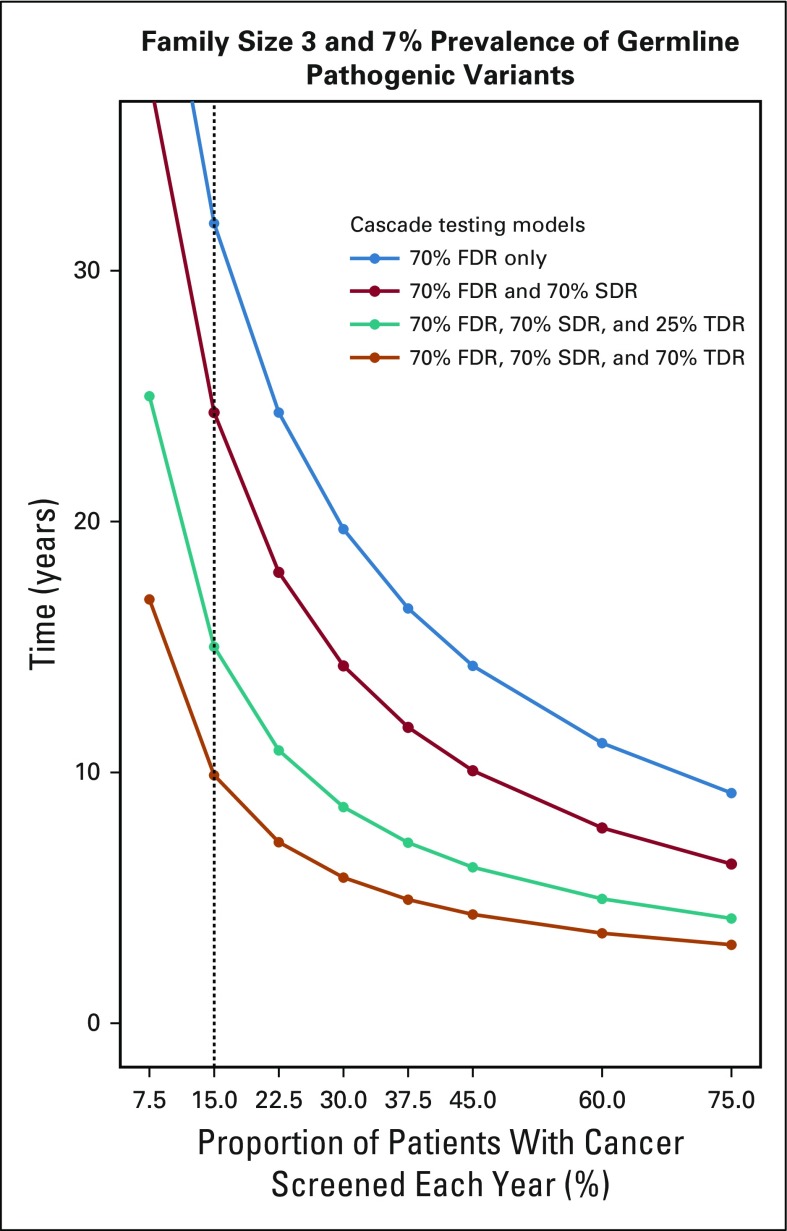
Sensitivity analysis of the example of a family structure of 3 siblings per generation and a 7% prevalence of pathogenic variants in 1 of 18 clinically actionable cancer susceptibility genes; Proportion, of 1.7 million incident cancers sequenced; Years to detect 3.9 million mutation carriers. FDR, first-degree relatives; SDR, second-degree relatives; TDR, third-degree relatives.

Figure 2 and the Data Supplement show the time to detect all cancer predisposing pathogenic variants of 18 cancer susceptibility genes in the US population, assuming a 5%-15% prevalence of germline variants in cohorts with cancer, varying proportions of family sizes, varying proportions of incident patients with cancer germline sequenced each year, and 0% to 75% rates of cascade of mutation detection to FDR, SDR, and TDR. Sensitivity analysis using a multiple regression model demonstrates that the time to detect pathogenic variants in the population is significantly affected by rates of FDR, SDR, and TDR tested by cascade, family size, and prevalence of mutations in patients with cancer, followed by the proportion of patients with cancer tested; Table 2 and Figure 1 demonstrate model fit metrics for the simulated data. As a base case, assuming a 7% frequency of clinically actionable germline pathogenic variants in 18 cancer susceptibility genes across all cancer types, based on a SEER weighted adjustment of data derived from patients tested at our Center (Data Supplement), and assuming 15% of incident cancers (the proportion of patients treated at US Comprehensive Cancer Centers) receiving tumor-normal or germline panel testing, for an average family size of 3 offspring per generation (as measured empirically; Data Supplement), the time to detect 3.9 million individuals with germline cancer susceptibility mutations would be 46.2, 22.3, 13.6, and 9.9 years, if 10%, 25%, 50%, and 70%, respectively, of FDR, SDR and TDR were tested for the familial mutation. In such a scenario (Fig 3), the 9.9-year interval to detect all mutations in the population compares with 59.5 years if there is no cascade testing of relatives (Data Supplement). The time to detect all mutations in the population decreased from more than 30 years if only 70% of FDR relatives are tested, to 15 years if 70%, 70%, and 25% of FDR, SDR, and TDR, respectively, are tested and 9.9 years if the proportion of TDRs tested increases from 25% to 70% (Fig 4).

**TABLE 2.**
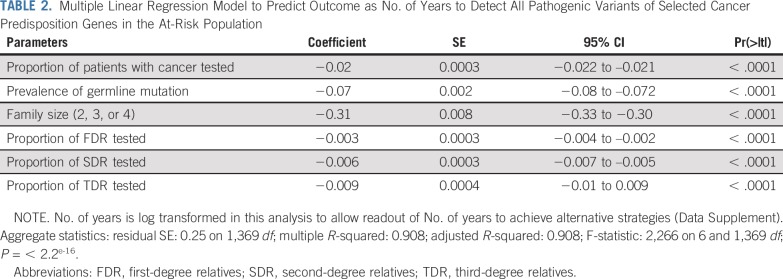
Multiple Linear Regression Model to Predict Outcome as No. of Years to Detect All Pathogenic Variants of Selected Cancer Predisposition Genes in the At-Risk Population

## DISCUSSION

Although referral rates for genetic testing have increased in the United States since 2004,^25-28^ only a modest proportion of individuals carrying pathogenic variants of common cancer susceptibility genes have been identified.^29^ Of 1.4 million breast cancer survivors eligible for genetic testing and at risk for subsequent cancers, 1.2 million have yet to be tested. An additional 10.7 million women who are cancer free but at risk for a primary cancer have not yet received genetic testing.^2^ For the most common form of hereditary colorectal cancer,^30^ it has been estimated that 98% of those carrying genetic variants predictive of Lynch syndrome have yet to be diagnosed,^31,32^ and DNA testing for other cancer syndromes is also less than optimal.^33,34^ These shortfalls are because of varying clinical guidelines as well as disparities in access to cancer prevention services.^35-41^ Even if universally accessible, it is now evident that 26%-56% of individuals with inherited pathogenic variants in cancer susceptibility genes will not be detected by clinical guidelines based on family history; this has been shown for “panel” genetic testing,^20^ as well as family history–targeted testing for *BRCA1/2, RET, FH, BAP1, VHL, MET, SDHA*, and *SDHB* germline mutations.^17,18,42-49^

Despite calls for population screening for common cancer predisposition genes such as *BRCA1/2*,^6^ and other high penetrance genes,^7^ there remain concerns about accessibility to diverse populations, including needs for intensive counseling and evaluation after detection of variants of unknown significance (VUS)^15,16^ and adverse psychological sequelae.^50^ For these and other reasons, the US Preventive Services Task Force in February 2019 reinforced recommendations against population-based genetic screening for *BRCA* mutations.^51,52^ The cost effectiveness of population sequencing depends on assumptions of the models^53,54^ reflecting varying costs of systems for monitoring and reclassifying VUS, raising public/health professional awareness, education, cost of medical interventions, delivery logistics, quality control, call-recall mechanisms, and fail-safe checks/processes for quality assurance.^54^ To provide *BRCA1/2* tests for all women older than 30 years of age in the United States would cost more than $14 billion,^55^ mostly as a nonreimbursable charge within the current US medical system.^56^ The model proposed here combines 2 elements as an alternative to population-based testing: peridiagnostic cancer genetic testing and familial cascade of results.

Genetic testing at the time of diagnosis of cancer (referred to by some as “mainstreaming”^57^) has several advantages.^20^ It identifies those who may benefit from poly (ADP-ribose) polymerase inhibitors for treatment of breast, ovarian, prostate, and other cancers^58-61^; checkpoint blockade immunotherapy for those with mismatch repair deficiency^62^; or targeted treatment of those with inherited mutations in *MTOR*,^63,64^ *RET*,^65^ *Hedgehog*,^66^ or other pathways. We have reported that the prevalence of pathogenic variants in cancer predisposition genes is higher in patients with metastatic prostate and other malignancies, compared with those with localized forms of these diseases.^20,61^ Germline testing of those with advanced malignancies regardless of family history also offers potential cost advantages, shown in the Data Supplement.

A limitation of the peridiagnostic testing model proposed is that it assumes sequencing of incident patients with cancer only. In 2016, there were 15.5 million individuals with a previous diagnosis of cancer (prevalent patients), including 429,000 survivors of childhood cancer in the United States.^21^ Although such individuals would eventually be tested by cascade, active ascertainment, and testing, even a fraction of these prevalent patients will decrease the time to achieve population-wide detection of cancer-predisposing mutations. Oversampling of early-onset or pediatric cancers will increase germline mutation detection and also lead to cascade testing of parents. Pediatric cancers demonstrate 8%-12% pathogenic germline variants,^22,67-70^ and there was a 2-fold increase in germline mutations in those older than 40 years of age compared with those younger than 60 years in 12,823 patients we have analyzed (MSK IMPACT^20^ and unpublished data). The model also assumes a low proportion of “de novo” mutations, observed for common syndromes^71^; however, when such de novo events occur, they will transmit to offspring, offering subsequent opportunity for cascade. Another limitation of the model is that it did not include polygenic effects of common, but lower-risk, single-nucleotide polymorphisms, which may offer the promise of passing thresholds of clinical actionability.^72^ The model was sensitive to the proportion of patients with cancer carrying germline pathogenic variants, which we have shown to be affected by founder mutations as well as case mix^20^; such correlations can be used to prioritize peridiagnostic genetic testing toward those in genetic isolates or with hereditary forms of cancer.

First described for cystic fibrosis and hypercholesterolemia^73-75^, cascade testing is a process of diffusion of genetic information from an affected family member (proband or index patient) to family members, involving iterative rounds of testing of both close and more distant relatives.^75^ Although 75%-82% of index patients may share familial cancer information with relatives,^76-78^ only 35%-64% of these at-risk adult relatives actually undergo genetic testing,^79-88^ with one study finding that 47.5% of FDR agreed to be tested after contact by a commercial laboratory offering them Internet access to self-pay sequencing for their familial mutation.^89^ Other investigators have increased familial testing to 46%-92% by contacting the proband’s relatives by mail or phone,^90-93^ achieving 57%-94% cascade to relatives in breast and colon cancer syndromes, respectively, with genetics professionals playing a role in the family outreach.^94-96^ Family dissemination of genomic risk information appears to be lower among minority populations,^97-99^ with patients of African American and Asian/Pacific ancestry less likely to share results with relatives.^81^ Because a focus on cascade testing could actually widen existing disparities in cancer genetic testing, a multifaceted approach will be required. Tailored and culturally sensitive interventions, including community-based outreach, partnering to decrease distrust and improve genetic literacy, navigators to assist genetic counselors and families, and an emphasis on telegenetics and phone counseling to decrease barriers, have been suggested.^100^ Our recent study in the United States by Frey et al^101^ (in this issue of *J Clin Oncol.*), a prior study in the United Kingdom,^96^ and a recent study in Trinidad and Tobago^102^ all achieved 60%-70% levels of cascade testing among at-risk relatives using a strategy of outreach by health care providers, indicating the cross-cultural feasibility of this approach. At the time of counseling, outreach to distant family members, such as first cousins, is vital; our analysis shows that expanding cascade from FDR to include SDR and TDR decreased *by half* the time to detect all carriers of pathogenic variants in the population. The model used was sensitive to family size; the estimates for family size were derived from empiric measures in a cancer clinic (Data Supplement). Population-based estimates of biologic family size are not included in household composition measures in census data; a recent population-based study found a mean of 19.7 FDR, SDR, and TDR per family sampled, with only modest variation by income strata.^23^ Finally, a substantial advantage of cascade-based approaches is decreased detection of VUS compared with population-wide sequencing. Cascade testing includes only established pathogenic variants that segregate in families, diminishing the complexities of counseling and interpretation of VUS.

Peridiagnostic and cascade testing has been shown to be cost effective for hypercholesterolemia^103^ and Lynch syndrome,^30^ where it is strongly influenced by the number of FDR tested.^104^ Importantly, the success of such strategies has been limited by the relative shortage of genetic counselors. As one means to address this need, digital media interventions to facilitate outreach of familial risk information have been piloted for colon cancer^99^ and pancreas cancer,^103^ and offer promise for implementing this proposed strategy. We have piloted digital learning tools in the context of a Web-based founder mutation testing program (www.bforstudy.com) that includes the option of follow-up with one’s primary health care provider.^105^ Integrating such tools in the peridiagnostic setting would offer at-home convenience and pretest education,^106,107^ and also avoid the logistic, privacy, and ethical barriers of “traceback” testing of DNA from paraffin archives of deceased patients.^108-111^ In contrast to population-screening approaches, patients with cancer can be offered germline genetic testing in a medical setting. The majority will want to know these results, emphasizing the potential advantages as well as the emotional and psychosocial components in a process that involves caregivers and family members.^112^

Testing for known inherited cancer susceptibility alleles in families is professionally endorsed,^113^ increasingly reimbursed by third-party carriers, and responsive to the legal and ethical “duty to warn” about familial disease risks.^114^ Although tumor genetic testing is now an Advanced Diagnostic Laboratory Test,^115^ Centers for Medicare & Medicaid Services is currently reevaluating including inherited germline testing in its coverage guidelines.^116^ These factors will help to mitigate disparities in access to genetic services, which constitute a barrier to peridiagnostic testing as well as cascade models.^113,117^ In the United States, peridiagnostic DNA sequencing has been initiated at many of the 71 National Cancer Institute (NCI)-designated cancer centers, which currently care for 15% of patients with cancer, and this effort could involve the more than 1,100 community cancer programs and oncology networks conducting clinical trials and approximately 250 academic and NCI-designated cancer research centers,^118,119^ with peridiagnostic genetic testing for prostate cancer to be offered through the US Veterans Administration.^120^ The Food and Drug Administration has approved targeting immunotherapy to a genetic defect in mismatch DNA repair,^121^ and a professional society recently provided a rationale for genetic testing of all patients with breast cancer.^116^

If adopted by clinicians, patients, and families, widespread peridiagnostic genetic testing and cascade to family members could identify all of the cancer predisposing mutations in the United States in a decade or less, diminishing the need for a costly effort to test and identify genetic variants in all healthy individuals in the population. As shown in recent studies (eg, Evans et al,^96^ Donenberg et al,^102^ and Frey et al^101^ [in this issue of *J Clin Oncol.*]), high rates of cascade of familial genetic information can be achieved by health care providers facilitating outreach and assisting patients and their families to communicate genetic risk information. Augmented by additional development, including Web-based applications, peridiagnostic cancer genetic testing, and familial transmission of genomic risk information can substantially decrease the burden of hereditary cancer.
